# Serum Calprotectin Levels and Inflammatory Biomarker Profiles in Adults with Culture-Confirmed Bacteremia

**DOI:** 10.3390/biology15201888

**Published:** 2026-10-20

**Authors:** Omer Faruk Duran, Ilkay Bahceci, Mehmet Kivrak

**Affiliations:** 1 Department of Medical Microbiology, Dr. Ersin Arslan Training and Research Hospital, Gaziantep 27090, Türkiye; omerfaruk.duran@hotmail.com; 2 Department of Medical Microbiology and Clinical Microbiology, Faculty of Medicine, Recep Tayyip Erdogan University, Rize 53100, Türkiye; ilkay.bahceci@erdogan.edu.tr; 3 Department of Biostatistics and Medical Informatics, Faculty of Medicine, Recep Tayyip Erdogan University, Rize 53100, Türkiye

**Keywords:** bacteremia, bloodstream infection, calprotectin, inflammatory biomarkers, host-response biomarkers

## Abstract

**Background/Objectives**: Circulating calprotectin reflects neutrophil and monocyte activation and has been proposed as a host-response biomarker in bacterial infection. However, its behavior in microbiologically confirmed bacteremia and its relationship with established inflammatory biomarkers remain incompletely defined. This exploratory study compared serum calprotectin and conventional inflammatory and hematological markers between adults with culture-confirmed bacteremia and controls without known comorbidities and examined calprotectin within the broader systemic inflammatory response associated with bacteremia. **Methods**: This prospective observational comparative study included 47 adults with culture-confirmed bacteremia and 30 controls without known comorbidities. Serum calprotectin, interleukin-6 (IL-6), C-reactive protein (CRP), procalcitonin, erythrocyte sedimentation rate (ESR), and hematological indices were compared between groups. Receiver operating characteristic (ROC) analyses were used descriptively to characterize between-group separation and were not intended to estimate clinical diagnostic accuracy. Hierarchical multivariable logistic regression was performed to assess the association between serum calprotectin and bacteremia after adjustment for age and sex. An extended model including the neutrophil-to-lymphocyte ratio (NLR), red cell distribution width–coefficient of variation (RDW-CV), and platelet count was considered exploratory. Gram-staining subgroups and 28-day outcomes were also examined. **Results**: Serum calprotectin concentrations were significantly higher in the bacteremia group than in controls (89.11 [80.16–95.35] vs. 62.90 [53.87–82.27] ng/mL; *p* < 0.001). Calprotectin showed an apparent area under the curve (AUC) of 0.840 (95% confidence interval [CI], 0.735–0.926), which was lower than those observed for IL-6, CRP, and procalcitonin. These ROC estimates reflect between-group separation within the present sample and should not be interpreted as estimates of diagnostic accuracy in patients with suspected infection. After adjustment for age and sex, each 10 ng/mL increase in calprotectin was associated with higher odds of belonging to the bacteremia group (adjusted odds ratio [aOR], 2.46; 95% CI, 1.58–3.85; *p* < 0.001). No biomarker significantly differentiated Gram-positive from Gram-negative bacteremia after false discovery rate (FDR) adjustment, and serum calprotectin was not associated with 28-day mortality. **Conclusions**: Serum calprotectin was elevated in adults with culture-confirmed bacteremia, consistent with activation of the systemic innate immune response. However, its apparent between-group discrimination was lower than that of IL-6, CRP, and procalcitonin, and it was not associated with Gram classification or 28-day mortality. Because the comparator group did not represent clinically similar patients undergoing evaluation for suspected infection, these findings should be interpreted as an exploratory characterization of the host response to bacteremia rather than as evidence of clinical diagnostic accuracy or added diagnostic value. Studies using clinically relevant comparator groups are needed to determine whether calprotectin provides incremental information beyond established biomarkers.

## Simple Summary

Bloodstream infections can become life-threatening very quickly, so doctors need reliable ways to recognize severe infection and understand how strongly the body is reacting. This study examined calprotectin, a protein released mainly by certain white blood cells during inflammation, in adults with confirmed bacteria in the bloodstream. We compared calprotectin with commonly used blood tests that reflect inflammation and also examined whether calprotectin levels differed according to the type of bacteria or survival at 28 days. Calprotectin levels were higher in patients with bloodstream infection than in the control group, showing that they reflect the body’s inflammatory response. However, several routine blood tests showed stronger separation between the groups, and calprotectin did not clearly distinguish different bacterial groups or predict 28-day survival. Because the control group was healthier than the patients typically evaluated for suspected infection, these results should be viewed as exploratory rather than as proof that calprotectin is a diagnostic test. The findings suggest that calprotectin may help researchers better understand the immune response to bloodstream infection, but further studies in real-world clinical populations are needed before it can be considered for routine patient care.

## 1. Introduction

Bloodstream infections (BSIs), including bacteremia, remain a major cause of severe infection, sepsis, and mortality worldwide and continue to represent an important clinical and public-health burden [1]. Early identification of the causative organism and timely administration of appropriate antimicrobial therapy are essential for improving clinical outcomes [2]. Blood culture remains the reference standard for diagnosing bacteremia because it provides both organism identification and antimicrobial susceptibility data. However, conventional blood-culture workflows require time for microbial growth, identification, and susceptibility testing, and cultures are generally incubated for up to five days before being reported as negative [3]. While awaiting definitive microbiological results, clinicians rely on clinical assessment together with routine inflammatory biomarkers to support early management decisions. Nevertheless, the performance of currently available host-response biomarkers remains variable across clinically heterogeneous populations [4,5,6,7].

Routine inflammatory biomarkers, including C-reactive protein (CRP), procalcitonin (PCT), interleukin-6 (IL-6), erythrocyte sedimentation rate (ESR), and leukocyte-derived indices, are commonly used to support early clinical decision-making. Their discriminatory performance, however, may vary according to sampling time, causative pathogen, infection source, and disease severity [8,9]. This variability has sustained interest in host-response biomarkers that may provide complementary information during bloodstream infection.

Calprotectin is a heterodimeric complex composed of the S100A8 and S100A9 proteins and is highly abundant in the cytosol of neutrophils, with lower expression in monocytes and macrophages. It contributes to innate immune defense through pro-inflammatory signaling and antimicrobial activity, including sequestration of transition metals required for microbial growth [10]. Elevated circulating calprotectin concentrations have been reported in bacterial infections and sepsis [10,11]. Recent reviews have highlighted the potential of circulating calprotectin as an inflammatory and infection-related biomarker while also emphasizing its overlap with established markers and its limited disease specificity [4]. A meta-analysis of critically ill patients reported pooled sensitivity and specificity of 0.77 and 0.85, respectively, for sepsis diagnosis, but substantial between-study heterogeneity was observed, underscoring the need for further validation in clinically representative populations [5].

Prospective studies in adults have likewise yielded heterogeneous findings. The CASCADE trial reported good discrimination of blood calprotectin for bacterial infection and an association with 30-day mortality [12]. In a prospective observational study of 391 adults presenting to the emergency department with possible sepsis, Christensen et al. reported an AUC of 0.777 for calprotectin in identifying infection; however, CRP (AUC, 0.913) and IL-6 (AUC, 0.895) showed better discrimination, while PCT had an AUC of 0.838 [6]. More recently, another prospective study found limited ability of plasma calprotectin to distinguish infection from non-infectious inflammation across heterogeneous clinical settings [7]. These inconsistencies highlight the importance of study population and comparator selection when evaluating calprotectin. Recent evidence from clinically comparable patients with suspected infection suggests that discriminatory performance may be more modest than that observed in case–control comparisons using healthy or minimally symptomatic controls [13]. In addition, serum calprotectin is elevated across a broad range of infectious and non-infectious inflammatory conditions, limiting its disease specificity [14]. Together, these observations reinforce the need for validation in clinically representative populations and for direct assessment of incremental value beyond established biomarkers [13,14].

Accordingly, the primary aim of this prospective observational comparative study was to characterize serum calprotectin concentrations in adults with culture-confirmed bacteremia relative to controls without known comorbidities and to examine these findings alongside established inflammatory and hematological markers. Because the comparator group did not represent patients undergoing evaluation for suspected infection, the study was not designed to estimate clinical diagnostic accuracy or to determine whether calprotectin can distinguish bacteremia from clinically similar febrile conditions. Secondary exploratory analyses characterized between-group separation using ROC analysis, compared biomarker concentrations between Gram-positive and Gram-negative bacteremia, and examined associations with 28-day mortality. Although the clinically relevant question is whether calprotectin provides additional information in patients already undergoing evaluation for suspected bloodstream infection, data describing calprotectin specifically in culture-confirmed bacteremia remain limited. The present study was therefore undertaken as an exploratory first step to characterize the biomarker profile of confirmed bacteremia rather than to establish clinical diagnostic utility.

## 2. Materials and Methods

**Study Design and Ethical Approval:** This prospective observational comparative study was conducted at Recep Tayyip Erdoğan University Training and Research Hospital, Rize, Türkiye. Adults with culture-confirmed bacteremia who were treated in inpatient wards or intensive care units were included in the bacteremia group. Adults without known comorbidities who attended the internal medicine outpatient clinic were enrolled as controls. Because the control group consisted of adults without known comorbidities rather than patients undergoing blood-culture testing for suspected infection, the study design was considered suitable for exploratory biomarker comparisons but not for direct validation of clinical diagnostic accuracy in a suspected-infection population. Accordingly, ROC analyses were interpreted as measures of apparent discrimination within the study sample rather than as estimates of diagnostic accuracy applicable to patients presenting with suspected bloodstream infection. The study protocol was approved by the Non-Interventional Clinical Research Ethics Committee of Recep Tayyip Erdoğan University Faculty of Medicine on 15 February 2023 (approval no. 2023/28). The study was conducted in accordance with the Declaration of Helsinki, and written informed consent was obtained from all participants before enrollment.

**Study Population and Eligibility Criteria**: Adult patients with bacteremia who were treated in the inpatient wards or intensive care units of Recep Tayyip Erdoğan University Training and Research Hospital between 15 February and 30 April 2023 were evaluated for inclusion. The bacteremia group included patients aged ≥18 years with bacterial growth considered clinically consistent with true bacteremia. Patients younger than 18 years, pregnant patients, and those with known chronic inflammatory diseases were excluded. Chronic inflammatory diseases included conditions such as vasculitis, rheumatoid arthritis, systemic lupus erythematosus, and glomerulonephritis, in which circulating calprotectin concentrations may be elevated independently of acute bacteremia. Pregnant patients were excluded because pregnancy is associated with physiological immunological and hematological changes that may independently influence inflammatory biomarkers and blood-cell indices. These exclusions were intended to reduce potential confounding of serum calprotectin measurements.

Neutropenia was not a predefined exclusion criterion. Other chronic comorbidities were not used as exclusion criteria for the bacteremia group. The control group consisted of adults aged ≥18 years attending the internal medicine outpatient clinic who had no known chronic comorbidities; pregnant individuals were excluded. Controls were selected from ambulatory outpatients without clinical findings suggestive of acute infection, including fever, erythema, swelling, local warmth, or loss of function. ESR, CRP, and complete blood count, including leukocyte counts, were considered when available as part of routine clinical evaluation during control selection. Recent antimicrobial exposure was not a predefined eligibility criterion for the control group. Thus, eligibility criteria regarding comorbidity status differed between the bacteremia and control groups. The bacteremia group included patients aged ≥18 years with bacterial growth considered clinically consistent with true bacteremia.

**Figure 1:**
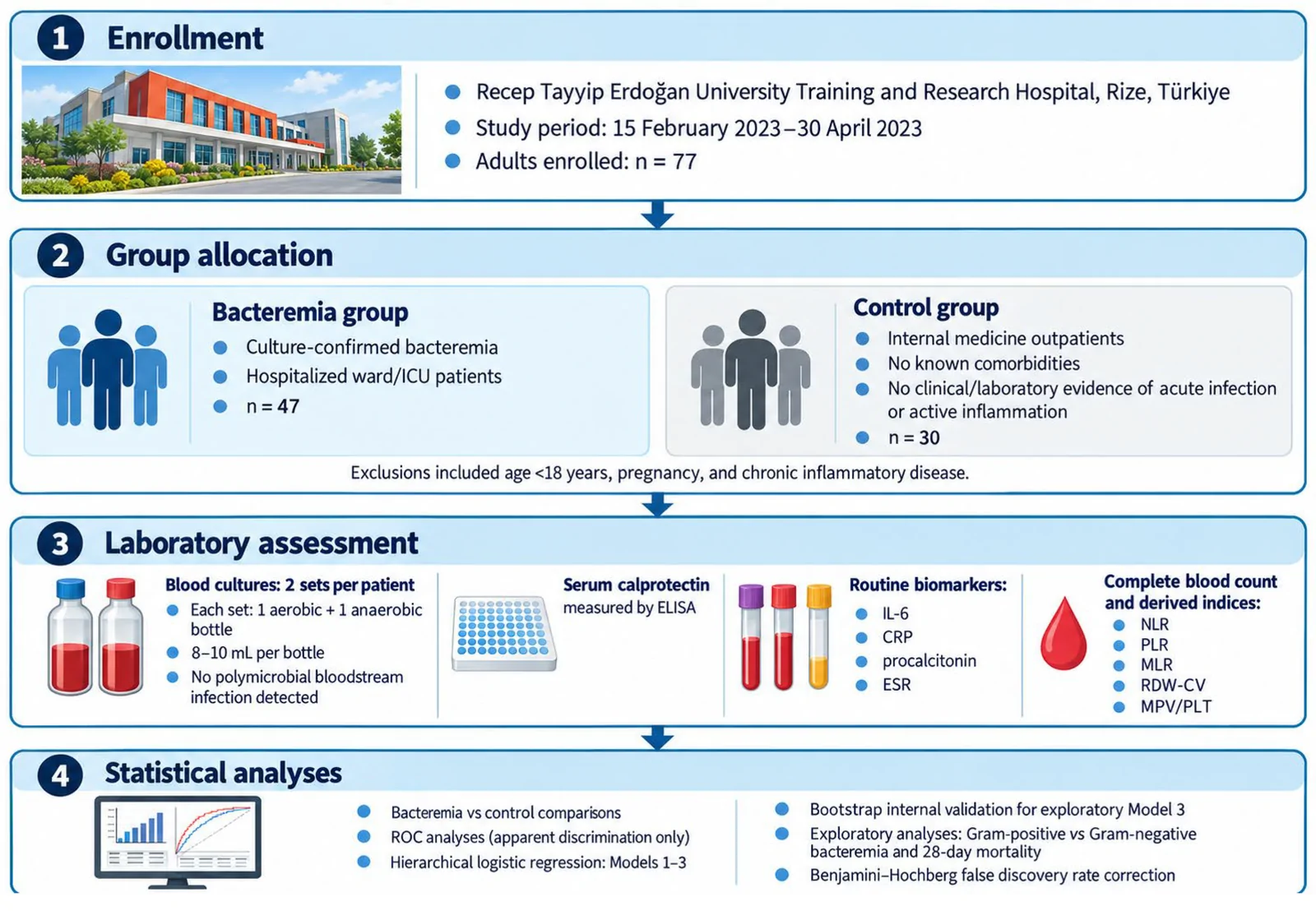
Study flow diagram and analytical workflow.

The minimum required sample size was calculated using G*Power version 3.1.9.7 for a two-group comparison of serum calprotectin concentrations. In the absence of directly comparable pilot data, a standardized effect size of 0.80 was assumed a priori, together with a two-sided type I error rate of 0.05 and 90% statistical power, resulting in a minimum required total sample size of 68 participants. Because an effect size of 0.80 represents a relatively large anticipated difference, this calculation was considered appropriate for the primary exploratory comparison rather than for definitive subgroup, prognostic, or multivariable analyses. Accordingly, the Gram-staining subgroup analysis, 28-day mortality analysis, and extended multivariable Model 3 were considered exploratory and were not independently powered for confirmatory inference. The final study population comprised 77 participants, including 47 patients with bacteremia and 30 controls. A detailed flowchart is presented in Figure 1.

**Definition of Bacteremia and Data Collection:** Bacteremia was defined as the isolation of a bacterial microorganism from blood culture. Only bacterial bloodstream infections were included; fungemia was outside the predefined scope of the study. Growth of microorganisms generally regarded as true pathogens was considered sufficient for inclusion when detected in at least one blood culture. For microorganisms commonly associated with skin microbiota, including coagulase-negative staphylococci, viridans group streptococci, diphtheroids, *Bacillus* spp., and *Propionibacterium* spp., growth in at least two separately collected blood-culture samples was required for classification as bacteremia.

Demographic data, including age and sex, and the inpatient treatment unit were recorded for each participant. Laboratory parameters obtained concurrently with blood-culture sampling included ESR, CRP, PCT, IL-6, and complete blood count parameters. Standardized baseline illness-severity scores, including SOFA, APACHE II, and qSOFA, were not available for systematic analysis. Detailed patient-level information on primary comorbidities, bacteremia source, acquisition setting, sepsis or shock status, and prior antimicrobial exposure was not systematically available for reliable retrospective analysis. The microorganisms isolated from blood cultures and 28-day survival status were retrieved from the hospital information management system. Gram-positive and Gram-negative bacteremia were classified according to the Gram-staining characteristics of the isolated microorganisms.

**Blood Culture Processing and Microorganism Identification:** Two sets of blood cultures were obtained from all patients with suspected bacteremia. Each set consisted of one aerobic and one anaerobic blood culture bottle. Approximately 8–10 mL of blood was inoculated into each bottle, corresponding to a total sampled blood volume of approximately 32–40 mL per patient. No polymicrobial bloodstream infections were identified in the study cohort. The bottles were incubated for up to five days using the Autobio BC120 automated blood-culture system (Autobio, Zhengzhou, China). Bottles generating a positive growth signal were subjected to Gram staining and subcultured onto 5% sheep blood agar, eosin methylene blue agar, and chocolate agar (RTA Laboratories, Kocaeli, Türkiye). The inoculated plates were incubated aerobically at 35–37 °C. After 24–48 h of incubation, bacterial colonies were identified using the VITEK 2 Compact automated system (bioMérieux, Marcy-l’Étoile, France). Antimicrobial susceptibility testing was performed using the same system, and results were interpreted according to the applicable European Committee on Antimicrobial Susceptibility Testing (EUCAST) criteria.

**Measurement of Routine Biomarkers and Hematological Parameters:** Routine inflammatory biomarkers and complete blood count parameters obtained concurrently with blood-culture sampling were recorded for each participant. Erythrocyte sedimentation rate (ESR) was measured using the Vision-C analyzer (YHLO Biotech, Shenzhen, China). Serum C-reactive protein (CRP) concentrations were measured using the AU5800 system (Beckman Coulter, Brea, CA, USA), procalcitonin (PCT) using the AQT90 FLEX analyzer (Radiometer, Brønshøj, Denmark), and interleukin-6 (IL-6) using the Cobas e601 system (Roche Diagnostics, Mannheim, Germany). Complete blood count parameters were measured using BC-6000 and BC-6200 hematology analyzers (Mindray, Shenzhen, China).

The evaluated hematological parameters included white blood cell, neutrophil, lymphocyte, monocyte, eosinophil, and basophil counts; red blood cell count; hemoglobin; hematocrit; mean corpuscular volume; mean corpuscular hemoglobin; mean corpuscular hemoglobin concentration; platelet count; mean platelet volume; plateletcrit; platelet distribution width; and red cell distribution width. The neutrophil-to-lymphocyte ratio (NLR) was calculated by dividing the absolute neutrophil count by the absolute lymphocyte count. The platelet-to-lymphocyte ratio (PLR) and monocyte-to-lymphocyte ratio (MLR) were calculated similarly. The mean platelet volume-to-platelet count ratio (MPV/PLT) was calculated by dividing mean platelet volume by platelet count. All laboratory measurements were performed in the hospital’s central laboratory using the corresponding automated analyzers.

**Serum Calprotectin Measurement:** For patients in the bacteremia group, serum samples were obtained concurrently with blood-culture collection. The available clinical records did not reliably establish whether empirical antimicrobial therapy had been initiated before sampling in all patients; therefore, pre-treatment status could not be systematically classified. For controls, serum samples were obtained at study enrollment.

Blood samples were collected into serum separator tubes (Vacusera, Türkiye) and allowed to clot for 30 min before centrifugation at 3500 rpm for 10 min using a Thermo Scientific™ Multifuge™ 3SR+ centrifuge (Thermo Fisher Scientific, Waltham, MA, USA). The separated serum was transferred into microcentrifuge tubes (AHN Biotechnologie GmbH, Nordhausen, Germany) and stored at −80 °C in an ultra-low-temperature freezer (New Brunswick Scientific, Canada) until analysis. Serum calprotectin concentrations were measured using a commercially available sandwich enzyme-linked immunosorbent assay kit (Human Calprotectin ELISA Kit, Elabscience (Houston, TX, USA), catalog no. E-EL-H2357) according to the manufacturer’s instructions.

The analytical measurement range of the assay was 1.56–100 ng/mL, with a reported sensitivity of 0.94 ng/mL. Samples exceeding the upper limit of the calibration curve were diluted 1:2 using the manufacturer-recommended sample diluent and re-assayed. Final concentrations were calculated by multiplying the measured value by the dilution factor. According to the manufacturer, intra-assay coefficients of variation ranged from 5.03% to 6.51%, and inter-assay coefficients of variation ranged from 6.95% to 8.99%.

Briefly, standards, blank controls, and serum samples were added to the designated wells and incubated at 37 °C for 90 min. After aspiration, biotinylated detection antibody was added, and the plate was incubated at 37 °C for 60 min. The wells were then washed three times, followed by the addition of horseradish peroxidase conjugate and incubation at 37 °C for 30 min. After five additional washing cycles, substrate solution was added, and the plate was incubated at 37 °C for 15–30 min. The reaction was terminated with stop solution, and optical density was measured at 450 nm using a Multiskan GO microplate spectrophotometer (Thermo Fisher Scientific, Waltham, MA, USA). Serum calprotectin concentrations were expressed as ng/mL.

**Statistical Analysis:** Statistical analyses were performed using jamovi version 2.7.38 and Python version 3.13.5 ROC analyses, bootstrap confidence intervals, hierarchical logistic regression models, and model-performance measures were calculated using the statsmodels version 0.14.6 and scikit-learn version 1.8.0 packages in Python.

The distribution of continuous variables was assessed using the Shapiro–Wilk test. Because most continuous variables were non-normally distributed or markedly skewed, they are presented as medians with interquartile ranges (IQR). Categorical variables are presented as numbers and percentages. Continuous variables were compared between two independent groups using the Mann–Whitney U test. Categorical variables were compared using Pearson’s chi-square test or Fisher’s exact test, as appropriate. Rank-biserial correlation was used as the standardized effect size for Mann–Whitney U comparisons. Positive rank-biserial correlation values indicated higher measurements in the bacteremia or Gram-positive group, depending on the comparison, whereas negative values indicated higher measurements in the reference group. Confidence intervals for rank-biserial correlations were estimated using 1000 bootstrap resamples. Odds ratios (ORs) with 95% confidence intervals (CIs) were reported for categorical comparisons.

To account for multiple testing, the Benjamini–Hochberg procedure was applied separately to the predefined families of comparisons involving bacteremia versus control groups, Gram-positive versus Gram-negative bacteremia, and 28-day survivors versus non-survivors. False discovery rate-adjusted *p*-values are reported as q-values. The 28-day mortality analyses were considered exploratory. Missing values were not imputed, and each analysis was performed using the available observations for the corresponding variable.

The apparent discriminatory performance of serum biomarkers and hematological indices for distinguishing patients with culture-confirmed bacteremia from controls was evaluated using receiver operating characteristic (ROC) curve analysis. Areas under the ROC curves (AUC) are presented with 95% CIs obtained using stratified bootstrap resampling. Statistical significance of each AUC was tested against the null value of 0.50. Optimal cut-off values were determined by maximizing the Youden index. Sensitivity and specificity are presented with exact Clopper–Pearson 95% CIs. Positive and negative likelihood ratios were calculated as sensitivity/(1—specificity) and (1—sensitivity)/specificity, respectively. When specificity was 100%, the positive likelihood ratio was considered infinite.

Because the cut-off values were derived and evaluated in the same study population, they were considered internally derived exploratory thresholds. Moreover, because the comparator group consisted of adults without known comorbidities rather than clinically suspected patients with negative blood cultures, the ROC-derived AUC, cut-off values, sensitivities, and specificities were interpreted as exploratory measures of apparent discrimination within the present comparative sample rather than as validated estimates of diagnostic accuracy applicable to patients presenting with suspected bloodstream infection.

Hierarchical multivariable binary logistic regression analysis was performed to evaluate the association between serum calprotectin and bacteremia after adjustment for demographic characteristics. Bacteremia status was entered as the dependent variable, with controls coded as 0 and patients with bacteremia coded as 1. Model 1 included age and sex, and Model 2 additionally included serum calprotectin. Model 2 was considered the primary adjusted model for evaluating the independent association between serum calprotectin and bacteremia.

An exploratory extended Model 3 additionally included NLR, RDW-CV, and platelet count to examine whether the association of calprotectin persisted after accounting for selected hematological markers. These variables were selected on the basis of clinical interpretability, representation of distinct hematological domains, and avoidance of mathematical redundancy. Neutrophil and lymphocyte counts were not entered together with NLR; platelet count was not entered together with PLR or MPV/PLT; and monocyte and lymphocyte counts were not entered together with MLR. IL-6, CRP, and procalcitonin were not included in the conventional multivariable models because their near-complete separation of the bacteremia and control groups could result in unstable coefficient estimates.

Given the modest sample size and number of predictors, Model 3 was considered exploratory and hypothesis-generating rather than a validated diagnostic prediction model. Age was scaled per 10-year increase, serum calprotectin per 10 ng/mL increase, and platelet count per 50 × 10^3^/µL increase. Because NLR showed a markedly right-skewed distribution, it was log_2_-transformed; the corresponding OR therefore represents the change in the odds of bacteremia associated with a two-fold increase in NLR. All three models were fitted using the same complete-case population to permit valid comparison between nested models.

Multicollinearity was assessed using variance inflation factors, and no problematic multicollinearity was detected, with all predictor-specific values below 2. Because the exploratory extended Model 3 included six predictors in a modest complete-case sample, internal validation was performed using bootstrap resampling to quantify model optimism. Bootstrap samples were generated with replacement, preserving the comparative structure, and the model was refitted in each resample. Optimism was estimated by comparing model performance in each bootstrap sample with performance obtained when the bootstrap-fitted model was applied to the original dataset. Mean optimism was then subtracted from the apparent AUC to obtain an optimism-corrected estimate. Bootstrap-corrected Brier score and calibration slope and intercept were also estimated. These analyses were intended to quantify potential overfitting rather than to establish external predictive validity.

Regression results are presented as ORs or adjusted ORs with 95% CIs. Model performance was summarized using apparent AUC, Nagelkerke R^2^, Akaike information criterion (AIC), and Brier score. For Model 3, bootstrap optimism-corrected AUC and Brier score, together with bootstrap calibration slope and intercept, were additionally reported as measures of internal validation. Lower AIC and Brier scores and higher AUC and Nagelkerke R^2^ values were considered indicative of better apparent model performance. Nested models were compared using likelihood-ratio tests. All tests were two-sided. A *p*-value < 0.05 was considered statistically significant for individual analyses, whereas a q-value < 0.05 was considered significant after false discovery rate adjustment.

## 3. Results

A total of 77 participants were included, comprising 47 patients with culture-confirmed bacteremia and 30 controls. Patients with bacteremia were older than controls (median age, 68.0 years (IQR, 58.0–81.0) vs. 61.5 years (IQR, 45.0–69.25); *p* = 0.013), whereas the sex distribution did not differ significantly between the groups (*p* = 0.380). Among patients with bacteremia, 24 (51.1%) had Gram-negative and 23 (48.9%) had Gram-positive bacteremia. Twenty-three patients (48.9%) were treated in the intensive care unit, and 24 (51.1%) died within 28 days. Among the 46 bacteremia patients with available absolute neutrophil counts, 8 (17.4%) had neutrophil counts < 1.5 × 10^3^/µL, including 2 (4.3%) with severe neutropenia (<0.5 × 10^3^/µL) (Table 1).

Of the 47 bacteremia cases, 24 (51.1%) were caused by Gram-negative microorganisms and 23 (48.9%) by Gram-positive microorganisms. *Escherichia coli* was the most frequently isolated Gram-negative microorganism, accounting for 8 of 24 Gram-negative cases (33.3%) and 17.0% of all bacteremia cases. *Staphylococcus aureus* was the most common Gram-positive microorganism, accounting for 9 of 23 Gram-positive cases (39.1%) and 19.1% of all bacteremia cases. *Acinetobacter baumannii* and *Enterococcus faecium* were the second most common Gram-negative and Gram-positive microorganisms, respectively (Table 2).

Serum calprotectin concentrations were significantly higher in patients with bacteremia than in controls (89.11 (IQR, 80.16–95.35) vs. 62.90 ng/mL (IQR, 53.87–82.27); *p* < 0.001), with a large standardized effect size (r = 0.681; 95% CI, 0.477–0.855). IL-6, CRP, procalcitonin, ESR, RDW-CV, NLR, PLR, MLR, and MPV/PLT were also significantly higher in the bacteremia group after correction for multiple comparisons. Conversely, lymphocyte and platelet counts were significantly lower in patients with bacteremia. Although neutrophil counts were modestly higher in the bacteremia group, total WBC counts did not differ significantly between groups (Table 3).

**Figure 2:**
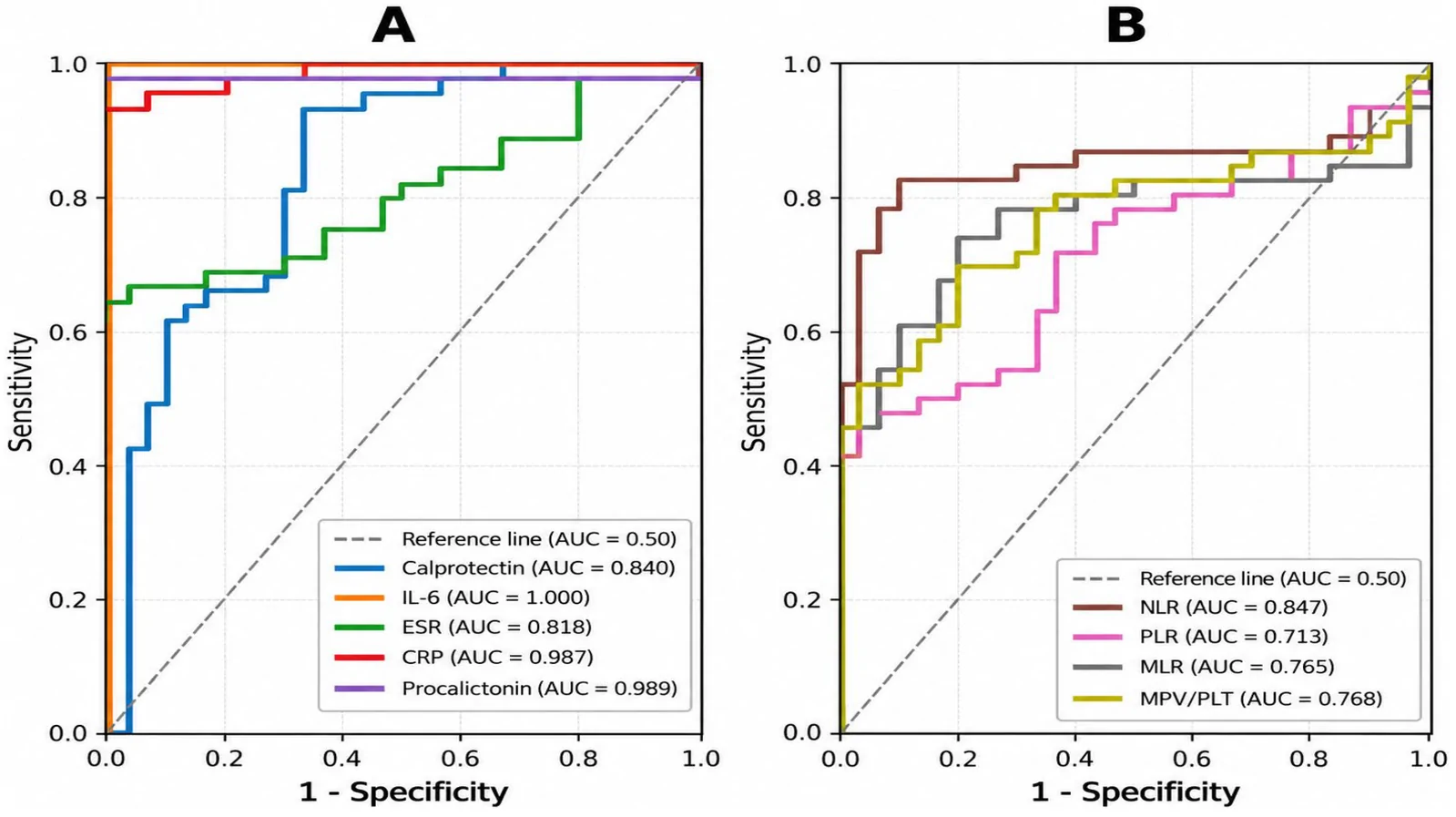
Receiver operating characteristic curves for distinguishing patients with culture-confirmed bacteremia from controls. Panel (**A**) shows the apparent discriminatory performance of serum calprotectin, interleukin-6 (IL-6), erythrocyte sedimentation rate (ESR), C-reactive protein (CRP), and procalcitonin. Panel (**B**) shows the performance of the neutrophil-to-lymphocyte ratio (NLR), platelet-to-lymphocyte ratio (PLR), monocyte-to-lymphocyte ratio (MLR), and mean platelet volume-to-platelet count ratio (MPV/PLT). Curves were generated from the available individual-level data for each marker. The dashed diagonal line represents discrimination no better than chance (AUC = 0.50). AUC, area under the receiver operating characteristic curve.

In ROC analyses, procalcitonin, CRP, and IL-6 showed the highest apparent discriminatory performance for distinguishing patients with bacteremia from controls, with AUC values of 0.989, 0.987, and 1.000, respectively. Serum calprotectin also demonstrated good apparent discrimination, with an AUC of 0.840 (95% CI, 0.735–0.926). At an optimal cut-off value of 70.72 ng/mL, calprotectin yielded a sensitivity of 93.6% and a specificity of 66.7%. Among the hematological indices, NLR showed the best discriminatory performance, with an AUC of 0.847 (95% CI, 0.746–0.931), 82.6% sensitivity, and 90.0% specificity at a cut-off value of 3.38 (Table 4 and Figure 2).

Hierarchical multivariable logistic regression analysis was performed to assess whether serum calprotectin was independently associated with bacteremia after adjustment for demographic characteristics. In Model 1, age was associated with bacteremia, with a 47% increase in the odds of bacteremia per 10-year increase in age (OR, 1.47; 95% CI, 1.09–1.97; *p* = 0.011). In the primary adjusted model (Model 2), the addition of serum calprotectin attenuated the association with age, whereas calprotectin remained strongly associated with bacteremia. Each 10 ng/mL increase in serum calprotectin was associated with 2.46-fold higher odds of bacteremia after adjustment for age and sex (aOR, 2.46; 95% CI, 1.58–3.85; *p* < 0.001). Model 2 showed better apparent model fit than Model 1, with an increase in apparent AUC from 0.683 to 0.871 and a significant likelihood-ratio test (*p* < 0.001). In the exploratory extended Model 3, which additionally included NLR, RDW-CV, and platelet count, serum calprotectin remained independently associated with bacteremia (aOR, 2.14 per 10 ng/mL increase; 95% CI, 1.17–3.91; *p* = 0.013). A two-fold increase in NLR was associated with higher odds of bacteremia (aOR, 2.10; 95% CI, 1.25–3.55; *p* = 0.005), whereas each 50 × 10^3^/µL increase in platelet count was associated with lower odds of bacteremia (aOR, 0.67; 95% CI, 0.47–0.94; *p* = 0.022). RDW-CV showed a borderline association (*p* = 0.054). Model 3 showed higher apparent performance measures than Model 2; however, bootstrap internal validation demonstrated measurable optimism, supporting cautious interpretation of this exploratory model (Table 5 and Table 6).

Bootstrap validation indicated measurable optimism in Model 3. The apparent AUC decreased from 0.946 to an optimism-corrected AUC of 0.913, and the Brier score increased from 0.090 to 0.121. A calibration slope of 0.64 indicated overfitting, although the calibration intercept remained close to zero (0.08). These findings support cautious interpretation of Model 3 as an exploratory model (Table 6).

Biomarker concentrations and hematological indices did not differ significantly between patients with Gram-positive and Gram-negative bacteremia, either before or after adjustment for multiple comparisons. Median serum calprotectin was numerically higher in Gram-positive bacteremia than in Gram-negative bacteremia (93.60 (IQR, 83.06–96.81) vs. 87.97 ng/mL (IQR, 77.65–93.34)), although the difference was not statistically significant (q = 0.625; 95% CI −0.094 to 0.562). Conversely, IL-6, procalcitonin, NLR, and MPV/PLT showed numerically higher median values in Gram-negative bacteremia, but their effect-size confidence intervals included zero (Table 7).

Among the 47 patients with bacteremia, 24 (51.1%) died within 28 days. Nominally higher IL-6 and MPV/PLT values and nominally lower lymphocyte, platelet, and ESR values were observed among non-survivors; however, these exploratory findings did not remain significant after correction for multiple comparisons. Serum calprotectin concentrations were comparable between survivors and non-survivors (88.21 (IQR, 79.85–94.75) vs. 89.50 ng/mL (IQR, 78.19–96.92); *p* = 0.853). CRP, procalcitonin, and NLR also did not differ significantly between the outcome groups (*p* > 0.05) (Table 8).

## 4. Discussion

In this prospective observational comparative study, serum calprotectin concentrations were significantly higher in adults with culture-confirmed bacteremia than in controls without known comorbidities, supporting an association with the systemic host response to bacteremia. However, the magnitude of between-group separation was lower for calprotectin than for IL-6, CRP, and procalcitonin. Because the comparator group was clinically dissimilar from the bacteremia group, the observed ROC characteristics should be interpreted as descriptive measures of separation within this study sample rather than as estimates of diagnostic accuracy. Accordingly, these findings do not establish that calprotectin can distinguish bacteremia from culture-negative sepsis, localized infection, viral infection, or non-infectious inflammatory disease, nor do they demonstrate incremental clinical value over established biomarkers. Calprotectin also did not differentiate Gram-positive from Gram-negative bacteremia and was not associated with 28-day mortality.

Calprotectin is a heterodimeric complex composed of S100A8 and S100A9 and is released predominantly by activated neutrophils and, to a lesser extent, monocytes and macrophages. It contributes to innate immune defense through inflammatory signaling and sequestration of transition metals required for microbial growth [10]. The marked increase in circulating calprotectin observed in patients with bacteremia is therefore biologically plausible. Previous studies have likewise reported elevated calprotectin concentrations during bacterial infection and sepsis [11,12,15,16]. In the prospective CASCADE trial, blood calprotectin showed good diagnostic performance for bacterial infection and was also associated with 30-day mortality [12]. Christensen et al., in a prospective observational study of 391 adults evaluated for possible sepsis in the emergency department, reported an AUC of 0.777 for calprotectin in identifying infection, compared with 0.913 for CRP, 0.895 for IL-6, and 0.838 for procalcitonin [6]. The AUC observed for calprotectin in our study (0.840) was somewhat higher but broadly comparable in magnitude. Importantly, Christensen et al. also found that CRP and IL-6 outperformed calprotectin, which is consistent with our finding that conventional inflammatory biomarkers, particularly IL-6, CRP, and procalcitonin, showed higher apparent AUC than calprotectin. Direct numerical comparisons should nevertheless be made cautiously because Christensen et al. evaluated patients with clinically suspected sepsis and adjudicated infection likelihood, whereas our study compared patients with culture-confirmed bacteremia with controls without known comorbidities. The lower discriminatory performance reported by Zimmermann et al. in clinically heterogeneous patients with suspected infection further illustrates the influence of study population and comparator selection on biomarker performance estimates [7]. These findings highlight the distinction between apparent discrimination in clinically dissimilar group comparisons and diagnostic accuracy in representative patient populations. Accordingly, the AUC of 0.840 observed for calprotectin in the present study should be regarded as a measure of apparent discrimination within this comparative sample rather than as a validated estimate of clinical diagnostic accuracy.

An important implication of our findings concerns the potential incremental clinical value of calprotectin. In the present cohort, calprotectin did not outperform established inflammatory biomarkers: IL-6, CRP, and procalcitonin showed substantially greater apparent between-group separation, whereas the routinely available NLR showed comparable performance. Calprotectin also failed to distinguish Gram-positive from Gram-negative bacteremia and was not associated with 28-day mortality. These observations are clinically relevant because conventional biomarkers and automated blood-cell counts are widely available, rapidly processed, and already integrated into routine laboratory workflows, whereas serum calprotectin in this study required a dedicated ELISA-based assay. Our findings therefore do not demonstrate a diagnostic or prognostic advantage sufficient to justify replacing or routinely supplementing established biomarkers with serum calprotectin. Its moderate specificity of 66.7% would also entail a substantial risk of false-positive classification if used as a standalone test. The present results therefore do not support the use of serum calprotectin to initiate, withhold, or de-escalate empirical antimicrobial therapy. Rather, calprotectin appears to function primarily as a marker of the inflammatory host response accompanying bacteremia. Whether it has a clinically useful niche remains uncertain and should be investigated in diagnostically challenging populations in which conventional biomarkers provide equivocal information. Such studies should specifically evaluate incremental value beyond established markers. Serial measurements may also be informative in determining whether calprotectin kinetics capture changes in host-response intensity or treatment response that are not reflected by a single baseline measurement. Until such evidence is available, serum calprotectin should be regarded as an exploratory host-response biomarker rather than as a routine clinical test for bacteremia.

The biological interpretation of circulating calprotectin also requires consideration of its limited pathogen specificity. Calprotectin (S100A8/A9) is abundant in neutrophils and may be released during neutrophil activation and neutrophil extracellular trap formation, suggesting that increased circulating concentrations can reflect neutrophil-driven inflammation and tissue stress rather than bacteremia specifically. This distinction is particularly relevant in acute viral infections. Calprotectin (S100A8/A9) is closely linked to neutrophil activation and NET formation [17]. Elevated circulating calprotectin has been reported in severe COVID-19 and has been associated with disease severity and adverse outcomes [18,19]. Severe influenza has likewise been associated with marked neutrophil activation and increased NET formation [20]. Studies of acute respiratory infections have also shown that calprotectin may be elevated in viral infections compared with healthy controls, although concentrations may be higher in bacterial disease [21]. Recent evidence from clinically comparable patients with suspected infection further suggests that the discriminatory performance of calprotectin is attenuated when comparator groups more closely reflect real-world diagnostic uncertainty [13]. In addition, recent evidence indicates that circulating calprotectin is elevated across a broad spectrum of infectious and non-infectious inflammatory conditions, thereby limiting its disease specificity [14]. Preanalytical and assay-related factors may also contribute to between-study variability, further emphasizing the need for standardized sampling and analytical procedures before calprotectin can be considered for routine clinical use. The elevated calprotectin concentrations observed in our bacteremia cohort should therefore not be interpreted as evidence of bacteremia-specific biological activity. Rather, calprotectin may primarily reflect the intensity of systemic neutrophil-driven inflammation. Because our control group did not include patients with acute viral infection or other inflammatory conditions, the present study cannot determine whether serum calprotectin reliably distinguishes bacteremia from severe viral respiratory infection. This question should be addressed in future prospective studies using clinically comparable febrile cohorts.

The composition of the control group is central to the interpretation of the present findings. Controls were adults without known comorbidities rather than symptomatic patients with suspected infection and negative blood cultures. The AUC obtained for calprotectin therefore reflects discrimination between culture-confirmed bacteremia and a relatively healthy outpatient population, rather than validation of its ability to distinguish bacteremia from clinically similar infectious or inflammatory conditions. This design may partly explain the clear separation in biomarker distributions and the very high apparent performance of conventional inflammatory markers. Accordingly, the reported AUC, sensitivities, specificities, and internally derived cut-off values were likely influenced by the marked clinical contrast between hospitalized bacteremia cases and relatively healthy outpatient controls. These estimates should not be generalized to real-world populations undergoing evaluation for suspected infection, in whom distinguishing bacteremia from non-bacteremic inflammatory illness is substantially more challenging. After adjustment for age and sex in the primary multivariable model, each 10 ng/mL increase in serum calprotectin was associated with approximately 2.5-fold higher odds of bacteremia. This association suggests that the observed relationship between calprotectin and bacteremia was not explained solely by differences in age and sex. Nevertheless, the finding demonstrates biological association and between-group separation rather than clinically actionable diagnostic utility.

The association between serum calprotectin and bacteremia also persisted in the exploratory extended model that additionally included NLR, RDW-CV, and platelet count. In this model, a two-fold increase in NLR was associated with higher odds of bacteremia, whereas higher platelet counts were associated with lower odds; RDW-CV showed a borderline association. However, Model 3 included multiple predictors in a relatively small comparative sample and was developed and evaluated in the same dataset. Its coefficients and apparent performance should therefore be interpreted cautiously and regarded as hypothesis-generating rather than as evidence of a validated diagnostic prediction model. Bootstrap internal validation supported this interpretation by demonstrating measurable optimism and a calibration slope below 1. The hematological findings are nevertheless consistent with the systemic response to severe bacterial infection. Patients with bacteremia had lower lymphocyte and platelet counts and higher neutrophil counts, RDW-CV, NLR, PLR, MLR, and MPV/PLT values than controls. Among the hematological indices, NLR showed the strongest apparent discrimination, with an AUC of 0.847, and remained independently associated with bacteremia in the extended model. NLR reflects the combination of neutrophil activation and relative lymphopenia associated with systemic inflammation. Similarly, thrombocytopenia may arise through platelet activation and consumption, endothelial interactions, and disseminated inflammatory processes. These derived indices, however, are mathematically dependent on their component blood-cell counts and should be interpreted as non-specific markers of systemic inflammation rather than as pathogen-specific indicators.

IL-6, CRP, and procalcitonin demonstrated almost complete separation between the bacteremia and control groups, with apparent AUC values of 1.000, 0.987, and 0.989, respectively, all exceeding that of calprotectin. These exceptionally high values should not be interpreted as evidence of near-perfect diagnostic accuracy in clinical practice, as they likely reflect the marked contrast between hospitalized patients with culture-confirmed bacteremia and controls without known comorbidities. Previous studies have reported higher IL-6 concentrations in bacteremic or microbiologically confirmed bacterial infections [22,23,24,25], while procalcitonin has shown diagnostic value for bacterial bloodstream infection and blood-culture positivity [26,27,28,29,30,31,32]. These findings further indicate that calprotectin did not outperform established inflammatory biomarkers in the present cohort.

CRP is a sensitive but non-specific acute-phase reactant that increases in both infectious and non-infectious inflammatory conditions [33,34]. Studies evaluating CRP in bacteremia have produced heterogeneous findings. CRP may show limited discriminatory performance during the early stages of febrile illness [35], and similar concentrations have been reported in septic patients with and without bacteremia [36]. Conversely, other studies have demonstrated associations between CRP and blood-culture positivity [30,32]. In the present study, CRP showed substantially greater apparent between-group discrimination than calprotectin. However, because the comparator groups were clinically dissimilar, this difference should be interpreted cautiously. IL-6, CRP, and procalcitonin were not included in the conventional multivariable regression models because their near-complete separation of the study groups could have produced unstable coefficient estimates.

ESR also showed good apparent discrimination, with an AUC of 0.818 and 100% specificity at the internally derived threshold. Previous studies have generally reported more modest performance. Chirouze et al. and van Nieuwkoop et al. observed limited or moderate discrimination in adults with fever or febrile urinary tract infection [26,27], while Leli et al. reported poor performance for ESR in predicting blood-culture positivity [28]. The higher performance observed in the present study is likely related to the use of controls without known comorbidities. ESR is also influenced by age, anemia, chronic inflammatory disorders, malignancy, and other non-infectious factors; therefore, it is unlikely to function as a sufficiently specific standalone biomarker in acute clinical practice.

The microbiological distribution of the cohort included a heterogeneous range of Gram-negative and Gram-positive pathogens, with *Escherichia coli* and *Staphylococcus aureus* being the most frequent isolates in their respective groups. This heterogeneity may have contributed to variability in host-response biomarker concentrations and limited organism-specific analyses. Because individual pathogen groups were small, the present study focused on the broader comparison of calprotectin between Gram-positive and Gram-negative bacteremia rather than organism-specific differences. Although the populations and clinical settings differed, the recurrence of these microorganisms emphasizes the potential influence of local microbiological ecology, antimicrobial resistance patterns, and the proportion of critically ill patients on the pathogen spectrum. Because the number of individual isolates was limited, reliable organism-specific biomarker analyses could not be performed in the present study.

No statistically significant differences in calprotectin or the other evaluated biomarkers were detected between Gram-positive and Gram-negative bacteremia after false discovery rate adjustment. Calprotectin was numerically higher in Gram-positive bacteremia, whereas IL-6, procalcitonin, NLR, and MPV/PLT were numerically higher in Gram-negative bacteremia; however, all effect-size confidence intervals included zero. Experimental evidence supports an important role for S100A8/S100A9-mediated responses in staphylococcal infection [37], but these biological mechanisms may not result in sufficiently distinct circulating calprotectin concentrations to differentiate Gram categories. Previous clinical studies have also reported inconsistent performance of routine biomarkers for distinguishing Gram-negative from Gram-positive bloodstream infection [8,29,31]. The lack of significant differences in the current study may additionally reflect the small subgroup sizes and considerable microbiological heterogeneity within each Gram-staining category. Interestingly, Christensen et al. reported significantly higher calprotectin concentrations in Gram-positive than in Gram-negative bacterial infections [6]. In our cohort, calprotectin showed the same numerical direction, with higher median concentrations in Gram-positive than in Gram-negative bacteremia (93.60 vs. 87.97 ng/mL), but the difference was not statistically significant after multiple-testing correction. The smaller subgroup sizes in our study and differences in infection definitions, disease severity, pathogen distribution, and sampling conditions may partly account for this discrepancy.

The prognostic findings differed from some previous reports. Elevated circulating calprotectin has been associated with mortality in critically ill and septic patients [15], hemodialysis patients with hyperphosphatemia [38], and patients with COVID-19 [18]. Larsson et al. also reported that calprotectin predicted 30-day mortality in intensive care patients and showed superior prognostic performance to procalcitonin [39]. In the present study, however, calprotectin concentrations were nearly identical in 28-day survivors and non-survivors. Nominal differences were observed for IL-6, ESR, lymphocyte count, platelet count, and MPV/PLT, but none remained statistically significant after correction for multiple comparisons.

Several explanations are possible. First, calprotectin was measured at a single time point in the present study. Therefore, we could not assess whether longitudinal changes in calprotectin paralleled clinical response, persistence of systemic inflammation, or subsequent deterioration. Serial measurements may be more informative than a single baseline concentration for evaluating the potential role of calprotectin as a dynamic host-response marker, and this possibility should be examined prospectively. Second, the bacteremia group represented a clinically severe population, with almost half of the patients treated in intensive care and approximately half dying within 28 days. This relatively restricted severity spectrum may have reduced discrimination between survivors and non-survivors. Third, mortality is influenced by numerous factors that were unavailable for adjusted analysis, including comorbidity burden, infection source, organ dysfunction, antimicrobial resistance, timing of effective treatment, and supportive-care interventions. Therefore, the absence of a significant association in this study should not be interpreted as definitive evidence that calprotectin has no prognostic value in bacteremia. Given the small number of bacteremia patients and deaths, together with the absence of adjustment for illness severity, comorbidity burden, infection source, acquisition setting, and antimicrobial treatment, the mortality analysis should be regarded as strictly exploratory and interpreted with particular caution.

The present study has several strengths. Bacteremia was microbiologically confirmed by blood culture, serum samples were collected concurrently with blood-culture sampling in the bacteremia group, and calprotectin was evaluated together with conventional inflammatory markers and multiple hematological indices. Effect sizes and confidence intervals were reported in addition to *p*-values, false discovery rate adjustment was used to address multiple comparisons, and the association between calprotectin and bacteremia was evaluated using hierarchical regression, with age and sex adjustment in the primary model and additional hematological variables examined in an exploratory extended model. The study also evaluated apparent discrimination, Gram-staining subgroups, and 28-day outcome within the same cohort.

Several limitations should also be acknowledged. First, this was a single-center study with a relatively small sample size. Although an a priori sample-size calculation was performed, the assumed standardized effect size of 0.80 was relatively large; therefore, the study may still have been underpowered for subgroup, prognostic, and multivariable analyses. In addition, recruitment was limited to 15 February–30 April 2023, and the study therefore did not capture a full annual cycle. Seasonal variation in respiratory infections, bloodstream infection epidemiology, and pathogen distribution may consequently have influenced the observed microbiological and biomarker patterns. Second, the bacteremia group primarily included hospitalized and critically ill patients, whereas the control group consisted of adults without known comorbidities. This contrast introduces spectrum and selection effects and probably overestimates the apparent discriminatory performance of several biomarkers. Future validation studies should include patients presenting with suspected infection, including culture-negative bacterial infection, localized infection, viral disease, and non-infectious inflammatory conditions. A more clinically informative design would enroll patients with suspected bloodstream infection at the time blood cultures are obtained and compare those with ultimately positive and negative cultures, while directly testing whether calprotectin adds discriminatory value beyond established biomarkers. Third, comorbidity-related eligibility criteria differed between groups: controls were required to have no known chronic comorbidities, whereas chronic conditions other than chronic inflammatory diseases were not exclusion criteria for the bacteremia group. The exclusion of patients with known chronic inflammatory diseases may further limit external validity, because many patients evaluated for possible bacteremia in routine practice have chronic inflammatory comorbidities, including chronic respiratory, rheumatologic, or gastrointestinal inflammatory disorders. Detailed information on comorbidity burden, infection source, acquisition setting, sepsis or shock status, acute organ dysfunction, antimicrobial exposure before sampling, and the interval between symptom onset and blood collection was unavailable for multivariable adjustment. Consequently, the extent to which these clinical factors contributed to the observed biomarker differences and high mortality could not be quantified. Standardized illness-severity scores such as SOFA, APACHE II, or qSOFA were also not available. Consequently, residual confounding by acute disease severity cannot be excluded, and the observed biomarker elevations may reflect both microbiologically confirmed bacteremia and the intensity of systemic inflammatory and organ-dysfunction responses. Fourth, biomarkers were assessed at a single time point; serial measurements might provide additional information regarding biomarker kinetics, treatment response, and prognosis. The study was restricted to bacterial bacteremia and did not include fungal bloodstream infections; therefore, the findings cannot be generalized to fungemia or other non-bacterial bloodstream infections. Fifth, the ROC-derived cut-offs and regression models were developed and evaluated in the same population and were not externally validated. Finally, the exploratory extended model and mortality analyses may have been underpowered.

## 5. Conclusions

Serum calprotectin was significantly elevated in adults with culture-confirmed bacteremia compared with controls without known comorbidities, consistent with its role as a marker of systemic innate immune activation. However, its apparent between-group discrimination was lower than that of IL-6, CRP, and procalcitonin, and calprotectin was not associated with Gram classification or 28-day mortality. Because the study compared hospitalized patients with confirmed bacteremia with a clinically dissimilar control group, it was not designed to evaluate the diagnostic accuracy of calprotectin in patients presenting with suspected infection or to establish incremental value over conventional biomarkers. The findings should therefore be regarded as exploratory characterization of the host-response profile of bacteremia. Future prospective studies should include clinically comparable patients with culture-negative sepsis, localized infection, viral illness, and non-infectious inflammatory conditions to determine whether calprotectin provides clinically meaningful information beyond established inflammatory markers.

## Figures and Tables

**Table 1:** Demographic and clinical characteristics of the study population. Data are presented as median (IQR) or n (%), as appropriate. Effect sizes are reported with 95% CI. Bold values indicate statistical significance at *p* < 0.05. **CI**, confidence interval; **IQR**, interquartile range.

Characteristic	Control Group (n = 30)	Bacteremia Group (n = 47)	Overall (n = 77)	Effect Size/95% CI	*p*-Value
Age, years, median (IQR)	61.5 (45.0–69.25)	68.0 (58.0–81.0)	67.0 (53.0–77.0)	r = 0.337 (0.087–0.569)	**0.013**
Sex, n (%)				OR = 0.66 (0.26–1.68)	0.380
Male	19 (63.3)	25 (53.2)	44 (57.1)		
Female	11 (36.7)	22 (46.8)	33 (42.9)		Reference
**Characteristic**				**Bacteremia Group (n = 47)**	
Gram-staining classification, n (%)					
Gram-positive bacteremia				23 (48.9)	
Gram-negative bacteremia				24 (51.1)	
Treatment unit, n (%)					
Inpatient ward				24 (51.1)	
Intensive care unit				23 (48.9)	
28-day outcome, n (%)					
Survived				23 (48.9)	
Died				24 (51.1)	

**Table 2:** Distribution of microorganisms isolated from blood cultures in patients with bacteremia. Data are presented as number and percentage. Percentages were calculated both within the corresponding Gram-staining group and among all patients with bacteremia (n = 47).

**Microorganism**	**n**	**Within Gram-Negative Group, %**	**Among All Bacteremia Cases, %**
*Escherichia coli*	8	33.3	17.0
*Acinetobacter baumannii*	7	29.2	14.9
*Stenotrophomonas maltophilia*	3	12.5	6.4
*Enterobacter cloacae*	1	4.2	2.1
*Haemophilus influenzae*	1	4.2	2.1
*Klebsiella pneumoniae*	1	4.2	2.1
*Pseudomonas aeruginosa*	1	4.2	2.1
*Salmonella* spp.	1	4.2	2.1
*Serratia marcescens*	1	4.2	2.1
**Total**	**24**	**100**	**51.1**
**Microorganism**	**n**	**Within Gram-Positive Group, %**	**Among All Bacteremia Cases, %**
*Staphylococcus aureus*	9	39.1	19.1
*Enterococcus faecium*	7	30.4	14.9
*Enterococcus faecalis*	2	8.7	4.3
*Streptococcus pyogenes*	2	8.7	4.3
*Streptococcus agalactiae*	1	4.3	2.1
*Streptococcus dysgalactiae*	1	4.3	2.1
*Streptococcus pneumoniae*	1	4.3	2.1
**Total**	**23**	**100**	**48.9**

**Table 3:** Comparison of biomarkers and selected hematological parameters between the bacteremia and control groups. Bold values indicate statistical significance at q < 0.05. **NLR**, neutrophil-to-lymphocyte ratio; **PLR**, platelet-to-lymphocyte ratio; **MLR**, monocyte-to-lymphocyte ratio; **MPV/PLT**, mean platelet volume-to-platelet ratio; **RDW-CV**, red cell distribution width–coefficient of variation; **ESR**, erythrocyte sedimentation rate; **CRP**, C-reactive protein; **IL-6**, interleukin-6; **CI**, confidence interval; **FDR**, false discovery rate; **IQR**, interquartile range; **WBC**, white blood cell count.

Parameter	Control Group, Median (IQR) (n = 30)	Bacteremia Group, Median (IQR) (n = 47)	Rank-Biserial Correlation, r (95% CI)	FDR-Adjusted q-Value
Calprotectin, ng/mL	62.90 (53.87–82.27)	89.11 (80.16–95.35)	0.681 (0.477–0.855)	**<0.001**
IL-6, pg/mL	2.94 (2.44–3.35)	175.20 (52.86–515.00)	1.000 (1.000–1.000)	**<0.001**
ESR, mm/h	6.50 (3.0–14.8)	35.0 (12.0–64.0)	0.636 (0.436–0.806)	**<0.001**
CRP, mg/L	4.65 (4.14–6.72)	171.00 (106.35–253.12)	0.975 (0.928–1.000)	**<0.001**
Procalcitonin, ng/mL	0.12 (0.12–0.12)	4.90 (0.79–21.00)	0.978 (0.933–1.000)	**<0.001**
WBC, ×10^3^/µL	6.75 (5.94–7.82)	8.45 (3.98–13.92)	0.096 (−0.162–0.354)	0.483
Lymphocyte count, ×10^3^/µL	2.29 (1.87–2.73)	0.59 (0.39–0.97)	−0.801 (−0.935–−0.632)	**<0.001**
Neutrophil count, ×10^3^/µL	3.83 (3.35–4.46)	6.69 (2.92–12.72)	0.290 (0.028–0.542)	**0.037**
Platelet count, ×10^3^/µL	244.0 (206.2–285.8)	144.5 (84.2–203.5)	−0.550 (−0.746–−0.325)	**<0.001**
RDW-CV, %	13.60 (13.20–14.10)	16.00 (14.83–17.08)	0.708 (0.504–0.881)	**<0.001**
NLR	1.83 (1.40–2.49)	10.28 (4.38–17.49)	0.694 (0.494–0.874)	**<0.001**
PLR	103.77 (91.11–156.72)	175.03 (117.46–413.75)	0.426 (0.184–0.638)	**0.002**
MLR	0.21 (0.17–0.27)	0.55 (0.27–0.92)	0.530 (0.297–0.741)	**<0.001**
MPV/PLT	0.042 (0.033–0.051)	0.083 (0.049–0.143)	0.536 (0.309–0.733)	**<0.001**

**Table 4:** Apparent discriminatory performance of serum biomarkers and hematological indices between adults with culture-confirmed bacteremia and controls without known comorbidities. **AUC**, area under the receiver operating characteristic curve; **CI**, confidence interval; **CRP**, C-reactive protein; **ESR**, erythrocyte sedimentation rate; IL-6, interleukin-6; **MLR**, monocyte-to-lymphocyte ratio; **MPV/PLT**, mean platelet volume-to-platelet count ratio; **NLR**, neutrophil-to-lymphocyte ratio; **PLR**, platelet-to-lymphocyte ratio; **LR+**, positive likelihood ratio; **LR−**, negative likelihood ratio.

Biomarker	AUC (95% CI)	Optimal Cut-Off	Sensitivity, % (95% CI)	Specificity, % (95% CI)	LR, +	LR, −	*p*-Value
Calprotectin, ng/mL	0.840 (0.735–0.926)	≥70.72	93.6 (82.5–98.7)	66.7 (47.2–82.7)	2.81	0.10	**<0.001**
IL-6, pg/mL	1.000 (1.000–1.000)	≥14.94	100.0 (92.5–100.0)	100.0 (86.8–100.0)	∞	0.00	**<0.001**
ESR, mm/h	0.818 (0.717–0.901)	≥25.00	64.4 (48.8–78.1)	100.0 (88.4–100.0)	∞	0.36	**<0.001**
CRP, mg/L	0.987 (0.965–1.000)	≥25.20	93.5 (82.1–98.6)	100.0 (88.4–100.0)	∞	0.07	**<0.001**
Procalcitonin, ng/mL	0.989 (0.967–1.000)	≥0.16	97.8 (88.2–99.9)	100.0 (88.4–100.0)	∞	0.02	**<0.001**
NLR	0.847 (0.746–0.931)	≥3.38	82.6 (68.6–92.2)	90.0 (73.5–97.9)	8.26	0.19	**<0.001**
PLR	0.713 (0.597–0.822)	≥206.06	45.7 (30.9–61.0)	96.7 (82.8–99.9)	13.7	0.56	**0.002**
MLR	0.765 (0.650–0.868)	≥0.30	73.9 (58.9–85.7)	80.0 (61.4–92.3)	3.70	0.33	**<0.001**
MPV/PLT	0.768 (0.657–0.868)	≥0.053	69.6 (54.2–82.3)	80.0 (61.4–92.3)	3.48	0.38	**<0.001**

**Table 5:** Hierarchical multivariable logistic regression models evaluating the association between serum calprotectin and culture-confirmed bacteremia. Bold values indicate statistical significance at *p* < 0.05. **aOR**, adjusted odds ratio; **AIC**, Akaike information criterion; **AUC**, area under the receiver operating characteristic curve; **CI**, confidence interval; **NLR**, neutrophil-to-lymphocyte ratio; **RDW-CV**, red cell distribution width–coefficient of variation.

Predictor	Model 1 aOR (95% CI)	*p*-Value	Model 2 aOR (95% CI)	*p*-Value	Model 3 aOR (95% CI)	*p*-Value
Age, per 10-year increase	1.47 (1.09–1.97)	**0.011**	1.34 (0.94–1.91)	0.108	1.17 (0.75–1.83)	0.491
Male sex	0.57 (0.21–1.54)	0.265	0.41 (0.12–1.45)	0.167	1.15 (0.21–6.46)	0.872
Calprotectin, per 10 ng/mL increase	—	—	2.46 (1.58–3.85)	**<0.001**	2.14 (1.17–3.91)	**0.013**
NLR, per two-fold increase	—	—	—	—	2.10 (1.25–3.55)	**0.005**
RDW-CV, per 1% increase	—	—	—	—	1.57 (0.99–2.48)	0.054
Platelet count, per 50 × 10^3^/µL increase	—	—	—	—	0.67 (0.47–0.94)	**0.022**
**Model characteristic**	**Model 1**		**Model 2**		**Model 3**	
Apparent AUC	0.683		0.871		0.946	
Nagelkerke R^2^	0.138		0.508		0.726	
AIC	99.8		74.2		57.6	
Brier score	0.216		0.138		0.090	
Likelihood-ratio test for nested models	Reference		*p* < 0.001 vs. Model 1		*p* < 0.001 vs. Model 2	

**Table 6:** Bootstrap internal validation of exploratory Model 3. A calibration slope below 1 indicates model overfitting. Model 3 was considered exploratory and hypothesis-generating rather than a validated clinical prediction model.

Model 3 Internal Validation Metric	Result
Complete-case sample	n = 77
Bacteremia events	47
Apparent AUC	0.946
Bootstrap optimism	0.033
Optimism-corrected AUC	0.913
Apparent Brier score	0.090
Optimism-corrected Brier score	0.121
Bootstrap calibration slope	0.640
Bootstrap calibration intercept	0.080

**Table 7:** Comparison of serum biomarkers and hematological indices between Gram-positive and Gram-negative bacteremia. **CI**, confidence interval; **CRP**, C-reactive protein; **ESR**, erythrocyte sedimentation rate; **FDR**, false discovery rate; **IL-6**, interleukin-6; **IQR**, interquartile range; **MLR**, monocyte-to-lymphocyte ratio; **MPV/PLT**, mean platelet volume-to-platelet count ratio; **NLR**, neutrophil-to-lymphocyte ratio; **PLR**, platelet-to-lymphocyte ratio; **WBC**, white blood cell count.

Parameter	Gram-Negative Bacteremia, Median (IQR) (n = 24)	Gram-Positive Bacteremia, Median (IQR) (n = 23)	Rank-Biserial Correlation, r (95% CI)	FDR-Adjusted q-Value
Calprotectin, ng/mL	87.97 (77.65–93.34)	93.60 (83.06–96.81)	0.246 (−0.094–0.562)	0.625
IL-6, pg/mL	285.20 (135.50–777.75)	123.00 (33.80–420.65)	−0.254 (−0.580–0.078)	0.625
ESR, mm/h	35.50 (15.00–59.50)	34.00 (10.00–65.00)	−0.006 (−0.356–0.336)	0.982
CRP, mg/L	147.40 (107.20–226.65)	191.10 (111.00–280.60)	0.104 (−0.229–0.433)	0.648
Procalcitonin, ng/mL	8.00 (1.35–21.75)	3.40 (0.64–16.00)	−0.140 (−0.464–0.204)	0.648
WBC, ×10^3^/µL	8.54 (4.70–14.06)	8.37 (3.44–12.98)	−0.096 (−0.425–0.251)	0.648
NLR	14.14 (6.03–18.90)	8.56 (3.74–14.77)	−0.191 (−0.516–0.146)	0.648
PLR	134.33 (105.56–287.70)	206.06 (127.38–437.42)	0.164 (−0.168–0.505)	0.648
MLR	0.38 (0.16–0.95), n = 23	0.62 (0.32–0.85), n = 23	0.229 (−0.127–0.546)	0.625
MPV/PLT	0.090 (0.049–0.161), n = 23	0.064 (0.047–0.132), n = 23	−0.115 (−0.448–0.229)	0.648

**Table 8:** Biomarker levels according to 28-day outcome among patients with bacteremia. **CRP**, C-reactive protein; **ESR**, erythrocyte sedimentation rate; **IL-6**, interleukin-6; **IQR**, interquartile range; **MPV/PLT**, mean platelet volume-to-platelet count ratio; **NLR**, neutrophil-to-lymphocyte ratio; **FDR**, false discovery rate.

Parameter	28-Day Survivors (n = 23), Median (IQR)	28-Day Non-Survivors (n = 24), Median (IQR)	*p*-Value	FDR-Adjusted q-Value
Calprotectin, ng/mL	88.21 (79.85–94.75)	89.50 (78.19–96.92)	0.853	0.853
IL-6, pg/mL	153.30 (34.77–237.40)	372.90 (117.07–4799.75)	**0.041**	0.074
ESR, mm/h	58.00 (26.00–73.00)	25.50 (7.50–42.25)	**0.024**	0.074
CRP, mg/L	139.40 (104.60–244.30)	173.85 (106.35–282.72)	0.655	0.842
Procalcitonin, ng/mL	3.40 (0.68–15.00)	6.10 (0.60–16.00)	0.798	0.853
Lymphocyte count, ×10^3^/µL	0.81 (0.47–1.40)	0.42 (0.32–0.80)	**0.012**	0.074
Platelet count, ×10^3^/µL	198.00 (90.10–283.00)	99.50 (61.50–184.50)	**0.041**	0.074
NLR	6.00 (3.37–15.96)	11.76 (6.11–29.53)	0.081	0.122
MPV/PLT	0.05 (0.03–0.11)	0.09 (0.05–0.21)	**0.027**	0.074

## Data Availability

The datasets used and/or analyzed during the current study are available from the corresponding author on reasonable request.

## Abbreviations

The following abbreviations are used in this manuscript:

AIC	Akaike information criterion
aOR	Adjusted odds ratio
AUC	Area under the receiver operating characteristic curve
BSIs	Bloodstream infections
CI	Confidence interval
CRP	C-reactive protein
ESR	Erythrocyte sedimentation rate
FDR	False discovery rate
IL-6	Interleukin-6
IQR	Interquartile range
LR+	Positive likelihood ratio
LR-	Negative likelihood ratio
MLR	Monocyte-to-lymphocyte ratio
MPV/PLT	Mean platelet volume-to-platelet count ratio
NLR	Neutrophil-to-lymphocyte ratio
OR	Odds ratio
PCT	Procalcitonin
PLR	Platelet-to-lymphocyte ratio
RDW-CV	Red cell distribution width–coefficient of variation
ROC	Receiver operating characteristic
WBC	White blood cell count
